# Supplementary material for: Content-rich biological network constructed by mining PubMed abstracts
Source: BMC Bioinformatics. 2004 Oct 8;5:147. doi: 10.1186/1471-2105-5-147 (PMC528731; doi:10.1186/1471-2105-5-147)
Supplement: Additional File 5 — The original Chilibot query results of the term "long-term potentiation (LTP)" and 22 other terms, limiting the latest references analyzed to the years 1990, 1995, 2000, and 2004. [file 1471-2105-5-147-S5.bz2 › chilibotAdditionalFile5/ltp2000/html/PKC_ARC.html]

 


 **PKC** and **ARC** 
  
Found 4 abstracts in PubMed,  **4 abstracts were retrieved and analyzed**.  


---

 Search Google  |
 PDF files only 
|  EDU domain only 

---

**Interactive relationship** (e.g. stimulation, inhibition, etc)

- In vitro studies in our laboratory have found that  **ARC**  increases 3H phorbol ester binding, an indirect measure of  **PKC**  translocation, and inhibits calcium buffering in microsomes and mitochondria.  Ref: 10022326 Toxicol Lett, 1998
- Recent experiments with rats found that developmental exposure to Aroclor 1254  **ARC**  affects the acquisition of a lever press response and produces long term changes in calcium buffering and protein kinase C  **PKC**  activity in the brain.  Ref: 10022326 Toxicol Lett, 1998

**Parallel relationship** (e.g. studied together, co-existance, homology, etc.)

- The present study examined protein kinase A PKA and protein kinase C  **PKC**  involvement in the maintenance of cellular tolerance to mu opioid receptor agonists resulting from chronic opiate exposure in neurosecretory cells of the hypothalamic arcuate nucleus  **ARC** .  Ref: 9618432 J Pharmacol Exp Ther, 1998
